# Atropine Premedication Facilitates Ultrasound-Guided Reduction by Saline Enema in Children With Intussusception

**DOI:** 10.3389/fphar.2019.00043

**Published:** 2019-01-31

**Authors:** Xiao Liu, Bei Xia, Hong-kui Yu, Lie-zhen Hu, Shu-min Fan, Dong Xiao, Li-xian Gu, Jia-kun Chen, Zhi-bo Wen, Xiao-peng Ma

**Affiliations:** ^1^Shenzhen Children’s Hospital, Shenzhen, China; ^2^Zhujiang Hospital, Southern Medical University, Guangzhou, China

**Keywords:** intussusception, atropine, enema, ultrasound, children

## Abstract

**Background and Objective:** Intussusception is the most frequent pediatric abdominal emergency. Intestinal spasm, ischemia, necrosis and even death may occur without prompt diagnosis and treatment. The ultrasound-guided reduction by saline enema is a preferred non-surgical procedure for intussusception. Muscular relaxants can relieve the intestinal spasm and edema by relaxing the intestinal smooth muscle, which may facilitate the treatment of intussusception. However, controversy persists on whether muscular relaxants are effective in the procedure. Therefore, the purpose of our study was to assess the efficacy of atropine known as a muscular relaxant in ultrasound-guided reduction by saline enema in children with intussusception.

**Methods:** All patients with intussusception diagnosed and treated in our department from July 2016 to February 2018 were included. Four hundred and thirty-seven children were enrolled and randomly divided into two groups: an atropine group and a control group. Intramuscular atropine at a dose of 0.02 mg per kilogram of body weight was administrated 15 min before ultrasound-guided reduction by saline enema in the atropine group. In the control group, the ultrasound-guided reduction was performed without using any muscular relaxants. The success rate, duration of the reduction, volume of saline, maximum intra-rectal pressure and complications were recorded and compared between the two groups.

**Results:** The success rate was 95.9% (212 out of 221) and 94.9% (205 out of 216) in the atropine group and the control group, respectively. No significant difference was observed in the success rate between the two groups (*P* > 0.05). The duration of reduction was significantly lower in the atropine group than in the control group (*P* < 0.01). The volume of saline was also significantly lower in the atropine group than in the control group (*P* < 0.05). The maximum intra-rectal pressure showed no difference between the two groups (*P* > 0.05).

**Conclusion:** Atropine premedication can facilitate ultrasound-guided reduction by saline enema in children with intussusception, by reducing the duration of reduction and the volume of saline in the procedure.

## Introduction

Intussusception is the most common cause of acute abdomen in children. The mean annual incidence worldwide varies from 0.24 to 2.4 per 1000 live births (Blanch et al., 2007; Gluckman et al., 2017). There is a high incidence in boys, and the male-to-female ratio is approximately 2:1 (Ko et al., 2007). The classic symptoms and signs include paroxysmal abdominal pain, vomiting, red jelly stool and a palpable abdominal mass (Lehnert et al., 2009; Territo et al., 2014). Accurate and timely diagnosis is imperative as a delay with longer duration of symptoms may result in intestinal ischemia, necrosis, perforation with peritonitis, higher rates of surgical intervention and rarely, death (Ito et al., 2012). Diagnosis based on clinical manifestations can be difficult sometimes due to the variability of its clinical manifestations. Ultrasound is currently the initial imaging modality in the diagnosis of pediatric intussusceptions with high sensitivity and specificity, 97.9 and 97.8%, respectively (Hryhorczuk and Strouse, 2009).

Intussusception can cause strangulated intestinal obstruction (Ito et al., 2012). It describes the process whereby a segment of bowel (the intussusceptum) telescopes into the lumen of the immediate distal segment (the intussuscipiens). The attached mesentery gets pulled along with the loop of bowel, resulting in constriction of venous outflow and impaired arterial perfusion. Intestinal spasm and ischemia remain continuously due to intestinal obstruction and impaired blood supply, eventually lead to intestinal necrosis and perforation.

Once the diagnosis of intussusception is established, the ultrasound-guided reduction by saline enema is a preferred non-surgical procedure with the advantages of lesser trauma, low cost and short hospital stay (Daneman and Navarro, 2004). The procedure involves introducing saline into the bowel, via the rectum, with a particular pressure that induces the bowel into its normal position. Most of the patients can be cured by saline enema reduction. But long duration of reduction and high hydrostatic pressure may cause further damage to the impaired intestine during the procedure, subsequently lead to intestinal perforation and other complications (Shehata et al., 2000; Bai et al., 2006).

The use of muscular relaxants might make the enema procedure faster and safer. Muscular relaxants can relieve the intestinal spasm and edema by relaxing the intestinal smooth muscle, which is beneficial to restore the blood supply. Recent animal studies have shown that scopolamine has a certain preventive effect on intussusceptions (Wetherell et al., 2007). However, there have been only a few reports on the use of atropine-assisted enema to improve the success rate (Grahl, 1983). At present, the efficacy of muscular relaxants is still controversial around the world, so it is seldom used in clinical practice (Ito et al., 2012). Therefore, the purpose of our study was to assess the efficacy of atropine known as a muscular relaxant in ultrasound-guided reduction by saline enema in children with intussusception.

## Patients and Methods

### Patients

Patients with intussusception diagnosed in our department from July 2016 to February 2018 were included. Intussusception was diagnosed according to the typical characteristic sonographic appearance of “target sign” in transverse view and “sleeve sign” in longitudinal view (Edwards et al., 2017). Inclusion criteria were: patients diagnosed with intussusceptions and treated by ultrasound-guided saline enema in our department. Exclusion criteria were: patients presented with clinical evidence of dehydration, shock, peritonitis or intestinal perforation, and suspected glaucoma patients. The ethics committee of Shenzhen children’s hospital approved the study and all parents of the patients gave written informed consent.

All patients were randomly divided into two groups: an atropine group and a control group. Fifteen minutes before reduction, an intramuscular atropine at the dose of 0.02 mg per kilogram of body weight was administrated and then ultrasound-guided reduction by saline enema was performed in the atropine group. In the control group, the ultrasound-guided reduction by saline enema was performed without using any muscular relaxants.

### The Protocol of Ultrasound-Guided Reduction by Saline Enema

The procedure including its necessity and risks was explained in detail to the parents. The materials for saline enema were prepared such as sufficient saline, a manometer, a tee pipe, an infusion rack, a Foleys catheter [16F] and a 50 ml syringe. The treatment room was equipped with an ECG monitor, central oxygen supply, a sphygmomanometer, etc., in case of emergency.

**FIGURE 1:**
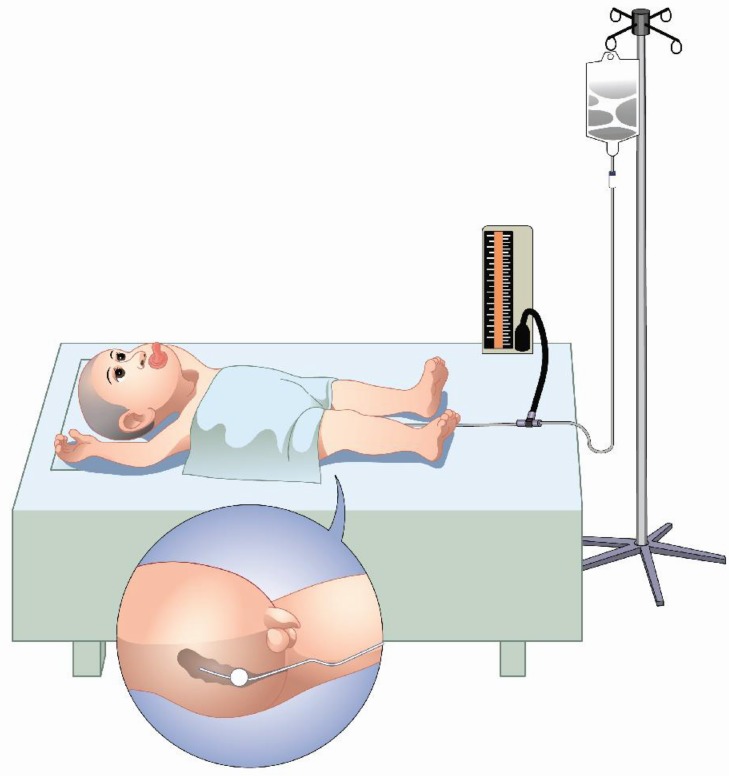
Illustration of the ultrasound-guided reduction by saline enema in children with intussusception.

A well-lubricated Foleys catheter [16F] was introduced per rectally for 7–10 cm. The bulb of the Foleys catheter was inflated with 30–50 ml of air and its position was confirmed by ultrasound. The catheter was connected to the tee pipe, another end of the tee pipe was connected to the manometer to measure the intra-rectal pressure, and the third end of the tee pipe was connected to the infusion bag containing warm saline (37–40°C). The infusion bag was kept at an extra height of 120 cm above the level of the bed. The parents kept company and held the child tightly on the thigh to seal the anus against leakage. The child was awake in a supine position whose condition was observed during the whole procedure (Figure 1).

**FIGURE 2:**
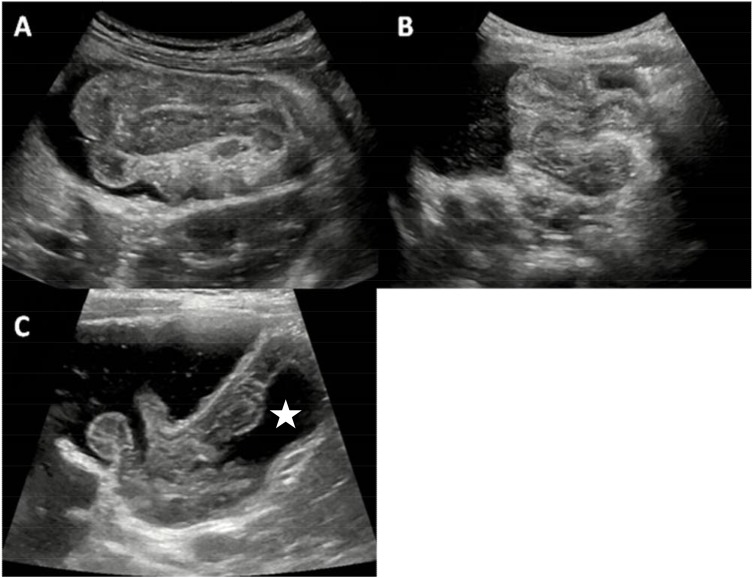
The ultrasonic manifestations during the ultrasound-guided reduction by saline enema. **(A)** Saline arrived at the intussuscipiens. **(B)** Intussusception gradually shortened. **(C)** Intussusception disappeared, saline passed through the ileocecal valve and fulfilled the terminal ileum (star).

When the patient was well prepared, the switch of the infusion device was turned on. Saline full filled the sigmoid colon, descending colon, transverse colon and ascending colon in sequence and arrived at the intussuscipiens (Figure 2A) under ultrasound monitoring (GE LogiQ E9, GE Co., Ltd., United States). Then *trans*-abdominal manual manipulation was performed to assist reduction by gently pressing abdomen counterclockwise to drive the liquid from left to right abdomen toward the intussuscipiens. In the meantime, the patients were instructed to mount a Valsalva maneuver. The above actions could be repeated for several times until the intussusception gradually shortened (Figure 2B) and disappeared (Figure 2C). During continuous ultrasound monitoring, underlying pathologic factors (lead points) in the intestinal wall and lumen were observed. If the intussusception could not be reduced in 30 min, the procedure was suspended. A repeated enema was performed after the patient took a rest for 30 min. The procedure could be repeated up to 3 times.

Criteria for successful reduction were disappearance of the intussusception and passage of fluid through the ileocecal valve (Figure 2C). Generally, the abdominal pain relieved and the child felt comfortable and became quiet at the same time.

During the procedure, the patients were monitored by ultrasound to observe whether there was presence of free peritoneal air or increased peritoneal fluid to exclude intestinal perforation. Once intestinal perforation occurred, the procedure should be stopped immediately, the fluid in the intestinal canal should be drained as far as possible, and the vital signs should be monitored. The patient was transferred to the operating room as soon as possible.

After successful reduction, all patients were admitted under observation and discharged when oral feeding and bowel movements were normal. All patients underwent unsuccessfully reduced intussusception were managed surgically.

Success or failure, duration of the reduction, volume of saline, maximum intra-rectal pressure and complications were recorded in each patient. The duration of the reduction included the time of saline infusion throughout the procedure. When the saline enema was repeated, the accumulated time is calculated as the duration of the reduction.

### Statistics Analysis

Measurement data were expressed as mean ± SD. Differences in duration of reduction, volume of saline and maximum intra-rectal pressure between the two groups were compared using unpaired Student’s *t*-test. The success rates and recurrent rates between the two groups were compared through χ^2^ test. The ultrasonic characteristics of intussusceptions, pathological lead points of secondary intussusceptions and unsuccessful factors of reduction between the two groups were also compared through χ^2^ test. Two-tailed *p*-values < 0.05 were considered statistically significant. All statistical analyses were performed using SPSS version 19.0 (SPSS, Inc., Chicago, IL, United States).

## Results

Generally, 437 patients were enrolled, of which 294 were male and 143 were female. The male-to-female ratio was approximately 2.06: 1. The median age of patients was 16 months (the youngest patient aged 2 months and the oldest aged 9 years). In our study, 92.2% (403 out of 437) of intussusceptions were ileocolic, 5.3% (23 out of 437) were colocolic, and the remaining 2.5% cases (11 out of 437) were ileoileocolic. Two hundred and twenty-one patients received atropine premedication before ultrasound-guided reduction by saline enema and 216 patients received the procedure without any muscular relaxants. There was no significant difference in age between the two groups (*P* > 0.05). The duration of symptoms was 3–72 h, with no significant difference between the two groups (*P* > 0.05). The size of the intussusceptions, including the length and the maximum external diameter, was not found to differ between the two groups (*P* > 0.05). There was also no significant difference in ultrasonic characteristics and specific classification of secondary intussusceptions between the two groups (*P* > 0.05). Main complaints and symptoms included 212 cases (48.5%) of paroxysmal abdominal pain, 207 cases (47.4%) of vomiting, 133 cases (30.4%) of excessive crying, 62 cases (14.2%) of bloody stool, 22 cases (5.0%) of abdominal mass, 15 cases (3.4%) of diarrhea, and 12 cases (2.7%) of fever. Background characteristics of patients are listed in Table 1.

**Table 1:** Background characteristics of patients in the two groups.

	The atropine group (*n* = 221)	The control group (*n* = 216)	*P*-value
Age (y)	1.83 ± 1.52	2.01 ± 1.62	0.256
Sex (Male/Female)	150/71	144/72	0.788
Duration of symptoms (hours)	22.19 ± 17.14	21.92 ± 15.81	0.867
Size of the intussusception			
Length (cm)	7.16 ± 2.10	7.18 ± 1.68	0.941
Maximum external diameterh (cm)	3.02 ± 0.37	3.03 ± 0.42	0.750
Ultrasonic characteristics			
Lymph node within the intussusception	45	48	0.636
Effusion inside the intussusception	15	13	0.743
Peritoneal fluid	36	43	0.326
Intestinal obstruction	3	5	0.455
Thicken intestinal wall of intussuscipiens	14	16	0.658
Lack of blood flow by color Doppler	4	3	0.726
Secondary intussusception			
Polyps	1	2	0.549
Meckel’s diverticulum	3	3	0.977
Intestinal duplication cyst	2	1	0.576
Lymphoma	1	1	0.987


**FIGURE 3:**
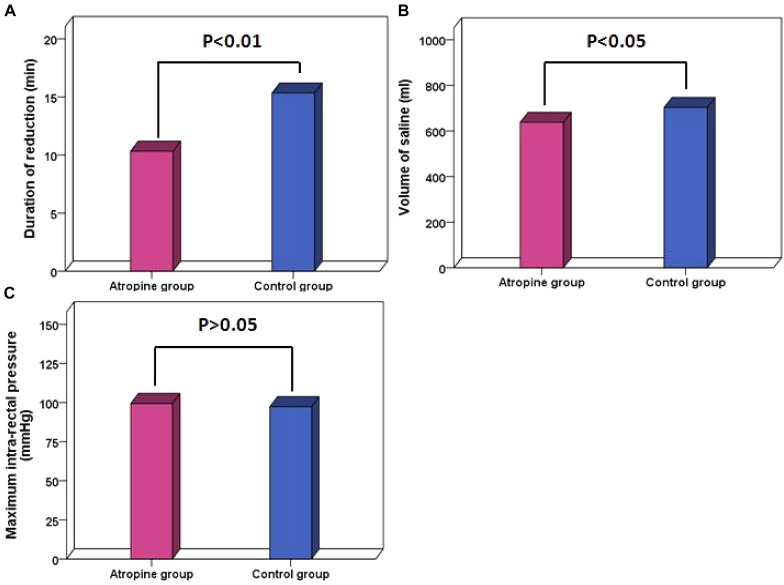
Parameters compared between the atropine group and the control group. **(A)** Duration of reduction. **(B)** Volume of saline. **(C)** Maximum intra-rectal pressure. *P* < 0.05 shows statistical significance.

The success rate was 95.9% (212 out of 221) and 94.9% (205 out of 216) in the atropine group and the control group, respectively. No significant difference was observed in the success rate between the two groups (*P* > 0.05). The duration of reduction was significantly lower in the atropine group than in the control group (*P* < 0.01) (Figure 3A). Volume of saline was also significantly lower in the atropine group than in the control group (*P* < 0.05) (Figure 3B). The maximum intra-rectal pressure showed no difference between the two groups (*P* > 0.05) (Figure 3C). In the control group, intestinal perforation occurred in one patient, presenting increased peritoneal effusion during the procedure. There was no significant difference in recurrence rates between the atropine group and the control group, which were 4.07 and 4.17%, respectively. Parameters of enema reduction and unsuccessful factors are listed in Table 2.

**Table 2:** Parameters of enema reduction and unsuccessful factors in both groups.

	Atropine group (*n* = 221)	Control group (*n* = 216)	*P*-value
Success rate	95.9% (212/221)	94.9% (205/216)	0.610
Time of reduction (min)	10.32 ± 8.89	15.34 ± 9.50	0.000
Volume of saline (ml)	637.96 ± 259.35	702.92 ± 342.73	0.026
Maximum intra-rectal pressure (mmHg)	99.30 ± 20.58	97.31 ± 17.04	0.273
Recurrence rate	4.07% (9/221)	4.17% (9/216)	0.960
Unsuccessful factors			
Ileoileocolic intussusception	4	4	0.974
Intestinal necrosis	3	3	0.977
Secondary intussusception	1	4	0.169
Deteriorated general condition	1	0	0.322


All 20 patients in whom enema reduction was unsuccessful underwent surgical reduction. Bowel resection was performed in six patients due to intestinal necrosis without lead points.

## Discussion

In the present study, our findings showed atropine premedication could significantly reduce the duration of reduction and the volume of saline in the ultrasound-guided reduction by saline enema in children with intussusceptions, and the success rate had no difference between the atropine group and the control group. To the authors’ knowledge, this is the first comparative study with large samples on the application of atropine known as a muscular relaxant for ultrasound-guided reduction by saline enema in children with intussusception.

The ultrasound-guided reduction of intussusception by saline enema has gradually become a worldwide-preferred non-surgical procedure for pediatric intussusception (Bai et al., 2006) since first described by Kim et al. (1982). Compared with air enema monitored by X-ray, the ultrasound-guided reduction by saline enema is visualized and more intuitive. It can visualize the various parts of the intussusception, showing possible pathological lead points such as polyps, Meckel’s diverticulum, lymphoma, etc. (Navarro et al., 2000). It’s radiation-free, avoiding the gonads’ exposure of children with long-term risk. Successful reduction can quickly relieve the child’s symptoms, avoiding surgery and complications such as surgery-related intestinal adhesion and infection. It is reported that the reduction rate of saline enema was similar to air enema, and complications such as perforation were rare, generally less than 1% (Daneman and Navarro, 2004). However, liquid reduction might associate with higher risk of peritoneal contamination compared with air enemas in the event of perforation (Carroll et al., 2017). All patients in our study were treated with saline enema reduction rather than air enema. From the results of our study, the success rate in the atropine group and the control group were 95.9 and 94.9%, respectively, both superior to the success rate of the previous literatures (Crystal et al., 2002; Flaum et al., 2016; Kanglie et al., 2018).

Reviewing the ultrasound characteristics, the unfavorable factors of unsuccessful reduction mainly included ultrasound findings of intestinal obstruction, peritoneal fluid, thicken intestinal wall of intussuscipiens, effusion inside the intussusception, and lack of blood flow by color Doppler in the intestinal wall of intussuscipiens (He et al., 2014; Gondek et al., 2018). The above ultrasound characteristics mainly caused by ileoileocolic intussusception, secondary intussusception, edema, necrosis and perforation of the intestinal wall according to surgery and pathologic findings, which were consistent with the literatures (Bramson and Blickman, 1992; Applegate, 2009). Therefore, it is necessary to consider the possibility of unsuccessful reduction and prepare for surgery in advance in the presence of the above findings by ultrasound. In our study, intestinal perforation occurred in one patient in the control group, presenting increased peritoneal fluid during the enema procedure.

Intussusception can cause strangulated intestinal obstruction (Ito et al., 2012). It describes the process whereby a segment of bowel telescopes into the lumen of the immediate distal segment. The attached mesentery gets pulled along with the loop of bowel, resulting in constriction of venous outflow and impaired arterial perfusion. With the persistent intestinal peristalsis, the intussusceptum continues to advance and cannot be reduced automatically. Intestinal spasm and ischemia remain continuously due to intestinal obstruction and impaired blood supply, eventually lead to intestinal necrosis, perforation and rarely, death. Prolonged enema procedure and excess pressure may increase the risk of perforation of the impaired intestine (Daneman et al., 1995).

The muscular relaxants can relieve the intestinal spasm and edema by relaxing intestinal smooth muscle, which may loosen the intussuscipiens, so that the intussusception reduction may be easier to achieve (Ito et al., 2012). So far, there are a few reports on the application of muscular relaxants in the reduction of intussusceptions. Three decades ago, Grahl (1983) summarized the experience of using scopolamine before enema, which is considered to improve the effect of enema. But this conclusion did not attract extensive attention. There is no consensus on the role of pharmacological adjuvants on enema reduction of intussusception (Gluckman et al., 2017). Therefore, they are not routinely used in clinical practice.

Atropine is a typical muscarinic cholinergic antagonist which can relieve spasms or cramps in the gastrointestinal smooth muscle, inhibit glandular secretion, expanding pupil, increasing intraocular pressure and regulating vision. It is clinically used to relieve visceral pain, including pain caused by gastrointestinal spasm, renal colic, biliary colic, gastric and duodenal ulcer. The blood concentration of atropine peaks 15–20 min after intramuscular injection. The effect lasts for 4–6 h with a half-life of 3.7–4.3 h. The metabolism of atropine is mainly through the hydrolysis of hepatocyte enzymes, and about 13–50% are discharged in the original form with urine within 12 h. In our study, the route of administration was intramuscular injection with a dose of 0.02 mg per kilogram of body weight. After intramuscular injection, atropine takes effect in 15–20 min. Therefore, the patient may show symptoms such as dry mouth and flushed face when the enema reduction gets started, indicating that the drug is effective. Small dose (less than 1 mg) of atropine has few side effects. Occasionally it may cause slow heart rate, slightly dry mouth and less sweat. Infants are more sensitive, so we need to focus on the heart rate especially for patients with brain injury. Atropine is banned for patients with glaucoma.

In this study, the duration of reduction and the volume of saline were significantly decreased and no complication of intestinal perforation occurred in the atropine group. We considered that atropine might play an important role since it can relax the intestinal smooth muscle and alleviate intestinal spasm and edema. It can also restore blood supply by loosening the intussuscipiens. Through the above mechanism, intussusception was easier and safer to be reduced. During the study period, we also found automatic reduction of intussusception occurred in three patients when we tried to perform the enema procedure after atropine administration, which might also benefit from the effect of atropine. The maximum intra-rectal pressure had no significant difference between the two groups, because it was determined by the height of the infusion bag, the pressure induced by *trans*-abdominal manual manipulation, and the pressure caused by children crying or mounting a Valsalva maneuver. Our results also indicated that the atropine group was easier and faster to achieve reduction under the same pressure compared with the control group. The recurrence rates in our study were 4.07% in the atropine group and 4.17% in the control group, which were consistent with previous study (Koh et al., 2006). Although atropine was seldom used in water enema, the application of atropine in barium enema could reduce the tension of colon, and aid comfort and reduce the duration of the enema procedure according to a previous study (Skucas, 1994).

Glucagon is another pharmacological adjuvant that can be used in intussusception according to previous studies. Glucagon is a hormone secreted by pancreatic islet α-cells and has a muscle relaxing effect. Early studies suggested that glucagon could improve the success rate of enema reduction (Ito et al., 2012), but a multicenter comparative study showed no significant effect on intussusception (Franken et al., 1983).

Sedatives are also applied in reduction of intussusception. Many sedatives have been reported in the literature, including diazepam, chloral hydrate, metazosing and morphine sulfate (Ito et al., 2012; van de Bunt et al., 2017). The use of sedatives or anesthesia during enema procedure can alleviate fear and pain. Children can calm down and be more cooperative. A study suggested that the success rate of reduction increased from 68.8 to 93.8% after the application of Midazolam with limited cases, only 16 cases in the atropine group and 16 cases in the control group (Eisapour et al., 2015). So far, there are few clinical studies support or oppose the use of sedatives. In a survey of European pediatric radiologists, only 34.9% of respondents routinely used sedatives for intussusception (Schmit et al., 1999). No sedatives were used in our study. We considered that children could be more initiative and cooperate with the parents’ company during the procedure. When the children were awake, the manometer showed increased intra-rectal pressure while they were crying or mounting a Valsalva maneuver which was conducive to reduction. There are also disadvantages to the use of sedatives including unpredictable complications, additional medical staff, inconvenience to observation of reaction and vital signs of children, and possible risks of allergic reaction such as shortness of breath (Mc and Rosenfeld, 2000).

The application of proper abdominal massage during the saline enema can promote reduction of intussusception. Grasso et al. (1994) reported that the success rate increased from 58 to 76% with the aid of transabdominal massage in air enema reduction. Real-time ultrasound monitoring revealed that *trans*-abdominal massage counterclockwise with the major thenar could promote the reduction. However, multiple repetitions are not recommended to avoid further damage to the intestinal wall of intussusception. If the enema procedure takes too long, the infusion should be suspended and the saline should be drained out if only a part of intussusception is retracted. Through the above operation, the intra-abdominal pressure reduced and the blood supply of intestinal wall may be restored. After an interval of 30 min, the saline enema could be performed again, which can increase the success rate of reduction and lower the risk of intestinal perforation. In this study, six patients underwent 2 or 3 enema procedures.

This article analyzed the role of atropine premedication in the ultrasound-guided reduction by saline enema through a comparative study of larger samples. However, there are certain limitations by ignoring other variables involved in the study, such as the skills of all the care team responsible for the reduction and characteristics of the patients can also be partly add to the duration of reduction and volume of saline.

## Conclusion

Atropine premedication can facilitate ultrasound-guided reduction by saline enema in children with intussusception by reducing the duration of reduction and the volume of saline in the procedure, which is beneficial to ease the suffering of children and lower the risk of complications. Therefore, we recommend the use of atropine prior to the reduction procedure.

## Author Contributions

XL wrote the paper and conceived and conducted the study. BX designed and improved the procedure of the technique and ensured its quality and safety as the ultrasonography department lead. H-kY wrote the paper and carried out the analysis. L-zH and S-mF implemented quality control of the procedure. DX provided valuable support in the therapeutic process. L-xG and J-kC contributed to data collection and management. Z-bW contributed to the discussion, supervised, and reviewed the text. X-pM promoted the clinical application of the technique. All authors read and approved the manuscript.

## Conflict of Interest Statement

The authors declare that the research was conducted in the absence of any commercial or financial relationships that could be construed as a potential conflict of interest.
