# Corrigendum: Atropine Premedication Facilitates Ultrasound-Guided Reduction by Saline Enema in Children With Intussusception

**DOI:** 10.3389/fphar.2019.00862

**Published:** 2019-08-02

**Authors:** Xiao Liu, Bei Xia, Hong-kui Yu, Lie-zhen Hu, Shu-min Fan, Dong Xiao, Li-xian Gu, Jia-kun Chen, Zhi-bo Wen, Xiao-peng Ma

**Affiliations:** ^1^Shenzhen Children’s Hospital, Shenzhen, China; ^2^Zhujiang Hospital, Southern Medical University, Guangzhou, China

**Keywords:** intussusception, atropine, enema, ultrasound, children

Xiao-peng Ma, Bei Xia, Lie-zhen Hu, Shu-min Fan and Dong Xiao were not included as authors in the published article. The corrected Author Contributions statement and Acknowledgments appear below. The authors apologize for this error and state that this does not change the scientific conclusions of the article in any way. The original article has been updated.

## Author Contributions

XL wrote the paper and conceived and conducted the study. BX designed and improved the procedure of the technique and ensured its quality and safety as the ultrasonography department lead. H-kY wrote the paper and carried out the analysis. L-zH and S-mF implemented quality control of the procedure. DX provided valuable support in the therapeutic process. L-xG and J-kC contributed to data collection and management. Z-bW contributed to the discussion, supervised, and reviewed the text. X-pM promoted the clinical application of the technique. All authors read and approved the manuscript.

